# N-3 Polyunsaturated Fatty Acids Decrease the Protein Expression of Soluble Epoxide Hydrolase via Oxidative Stress-Induced P38 Kinase in Rat Endothelial Cells

**DOI:** 10.3390/nu9070654

**Published:** 2017-06-24

**Authors:** Takashi Okada, Katsutaro Morino, Fumiyuki Nakagawa, Masashi Tawa, Keiko Kondo, Osamu Sekine, Takeshi Imamura, Tomio Okamura, Satoshi Ugi, Hiroshi Maegawa

**Affiliations:** 1 Department of Medicine, Shiga University of Medical Science, Shiga 520-2192, Japan; tokada@belle.shiga-med.ac.jp (T.O.); fumiyuki-nakagawa@cmicgroup.com (F.N.); sekine@belle.shiga-med.ac.jp (O.S.); sugi@belle.shiga-med.ac.jp (S.U.); maegawa@belle.shiga-med.ac.jp (H.M.); 2 Nishiwaki Laboratory, CMIC Biopharma Co., Ltd., Hyogo 677-0032, Japan; 3 Department of Pharmacology, Shiga University of Medical Science, Shiga 520-2192, Japan; tawa@belle.shiga-med.ac.jp (M.T.); timamura@med.tottori-u.ac.jp (T.I.); okamura@belle.shiga-med.ac.jp (T.O.); 4 Department of Public Health, Shiga University of Medical Science, Shiga 520-2192, Japan; kon@belle.shiga-med.ac.jp; 5 Division of Molecular Pharmacology, Department of Medicine, Tottori University, Tottori 683-8503, Japan

**Keywords:** soluble epoxide hydrolase, epoxyeicosatrienoic acid, p38 kinase, *n*-3 PUFA, endothelial function

## Abstract

*N*-3 polyunsaturated fatty acids (PUFAs) improve endothelial function. The arachidonic acid-derived metabolites (epoxyeicosatrienoic acids (EETs)) are part of the endothelial hyperpolarization factor and are vasodilators independent of nitric oxide. However, little is known regarding the regulation of EET concentration by docosahexaenoic acid (DHA) and eicosapentaenoic acid (EPA) in blood vessels. Sprague-Dawley rats were fed either a control or fish oil diet for 3 weeks. Compared with the control, the fish oil diet improved acetylcholine-induced vasodilation and reduced the protein expression of soluble epoxide hydrolase (sEH), a key EET metabolic enzyme, in aortic strips. Both DHA and EPA suppressed sEH protein expression in rat aorta endothelial cells (RAECs). Furthermore, the concentration of 4-hydroxy hexenal (4-HHE), a lipid peroxidation product of *n*-3 PUFAs, increased in *n*-3 PUFA-treated RAECs. In addition, 4-HHE treatment suppressed sEH expression in RAECs, suggesting that 4-HHE (derived from *n*-3 PUFAs) is involved in this phenomenon. The suppression of sEH was attenuated by the p38 kinase inhibitor (SB203580) and by treatment with the antioxidant N-acetyl-L-cysteine. In conclusion, sEH expression decreased after *n*-3 PUFAs treatment, potentially through oxidative stress and p38 kinase. Mild oxidative stress induced by *n*-3 PUFAs may contribute to their cardio-protective effect.

## 1. Introduction

Endothelial dysfunction causes atherosclerosis and is induced by various factors including smoking, hypertension, dyslipidemia, and diabetes mellitus [1]. Nitric oxide (NO), which is generated by endothelial NO synthase (eNOS), is a major modulator in acetylcholine-induced vasodilation and reactive hyperemia [2]. The endothelial-derived hyperpolarization factor (EDHF) is an alternative factor in this process, which was indirectly determined to be a non-NO/non-prostacyclin (PGI2) vasodilator (using an eNOS inhibitor and aspirin) [3]. Recent studies have revealed that in arteries from a variety of species, including humans, epoxyeicosatrienoic acids (EETs) act as EDHFs [4,5]. The endothelium expresses soluble epoxide hydrolase (sEH), which rapidly hydrolyzes EETs to dihydroxyeicosatrienoic acids (DHETs) [6]. Both the potentiation of vascular EET synthesis and the reduction in EET hydrolysis increase EET bioavailability, which results in decreased vascular tone. Currently, sEH inhibitors are potential candidates as novel anti-hypertension drugs [7].

Increased fish consumption or *n*-3 polyunsaturated fatty acids (PUFAs), including eicosapentaenoic acid (EPA) and docosahexaenoic acid (DHA), decreases the risk of cardiovascular disease [8,9]. Although decreased plasma triglycerides and anti-platelet and anti-inflammatory effects may play a significant role in this, the precise molecular mechanisms underlying this effect remain unknown [10,11]. Our group and others have found that endothelial dysfunction is ameliorated by intervention with *n*-3 PUFAs in both human studies [12,13,14,15] and animal models [16]. Increased eNOS expression was observed in the aortas of *n*-3 PUFA-fed rats [17]. However, the effect of *n*-3 PUFAs on the EDHF pathway remains unknown.

The aim of this study was to test the hypothesis that *n*-3 PUFAs increase EETs in the aorta by decreasing sEH expression. To this end, we measured the 4-hydroxy hexenal (4-HHE) and EET content, and tested their role in the aorta and cultured endothelial cells.

## 2. Materials and Methods

### 2.1. Reagents

Dulbecco’s modified Eagle’s medium (DMEM) and fetal bovine serum (FBS) were obtained from Life Technologies (Tokyo, Japan). EPA, DHA, and 4-HHE were purchased from Cayman (Ann Arbor, MI, USA). Anti-β-actin antibody (A5316) and N-acetyl-l-cysteine (NAC) were purchased from Sigma-Aldrich (St. Louis, MO, USA). Fatty acid-free bovine serum albumin (BSA) was purchased from Nacalai Tesque (Kyoto, Japan). Anti-epoxide hydrolase 2 (sEH) antibody was purchased from Gene Tex Inc. (Irvine, CA, USA). Anti-p38 kinase (p38) (#9212) and anti-phospho-p38 (#9211) antibodies were purchased from Cell Signaling (Danvers, MA, USA). Anti-PECAM-1 (M-20) antibody and anti-heme oxygenase 1 (HO-1) antibody were purchased from Santa Cruz (Dallas, TX, USA). Anti-cytochrome P450 2J2 (CYP2J2) antibody was purchased from Origene (Rockville, MD, USA). Horseradish peroxidase (HRP)-conjugated anti-mouse and anti-rabbit antibodies were purchased from Amersham Biosciences Corp. (Piscataway, NJ, USA). Small interfering RNA (siRNA) was purchased from Life Technologies (Tokyo, Japan). SB203580 was purchased from Calbiochem (Cambridge, UK). All other reagents and chemicals were from standard suppliers.

### 2.2. Animals

All animal experiments were approved by the Shiga University of Medical Science Committee on Animal Research. Male Sprague-Dawley (SD) rats were assigned to two groups: fish oil-fed (#112246: 27% (weight/weight) menhaden fish oil diet (59% fat-derived calories; 16% EPA, 9% DHA); Dyets Inc., Bethlehem, PA, USA) and soy oil-based control diet-fed (#110700, AIN-93 G). After 3 weeks, rats were euthanized by pentobarbital sodium overdose, to analyze the effect of each diet on the acetylcholine-dependent vasorelaxation reaction.

### 2.3. Endothelium-Dependent Vasorelaxation

The experimental procedure for artery strips was performed as previously reported, with minor modifications [18]. Briefly, 8-week-old male Sprague-Dawley (SD) rats were housed in an environmentally controlled room with a 12 h light/dark cycle and free access to food and water. Rats were fed a control or fish oil diet for 3 weeks. Rats injected with heparin (500 U/kg, iv) were euthanized by bleeding from the abdominal aorta under deep anesthesia. The thoracic aorta was dissected, excised and cut into strips with special care being taken to preserve the endothelium. The strips were then fixed vertically between hooks in a muscle bath (10 mL capacity), containing a modified Ringer-Locke solution bubbled with a gas mixture of 95% O_2_ and 5% CO_2_ (pH 7.4 at 37 ± 0.3 °C). The hook anchoring the upper end of the strip was connected to the lever of a force-displacement transducer (Nihon Kohden Kogyo Co., Tokyo, Japan). The resting tension was adjusted to 2.0 g, which is optimal for inducing maximal contraction. Before starting the experiments, all aorta preparations were equilibrated in the bathing medium for 60–90 min, during which time the solution was replaced every 10–15 min. The strips were partially contracted with phenylephrine (0.1 µM). After the contraction reached a plateau, a concentration–response curve for acetylcholine was obtained by adding the drug directly to the bathing medium in cumulative concentrations. At the end of each experiment, papaverine (0.1 mM) was added to induce maximal relaxation, which was taken as 100% for relaxation induced by the agonist. 

### 2.4. Cell Culture

Cultures were grown at 37 °C in a humidified atmosphere with 5% CO_2_. Rat aorta endothelial cells (RAECs) were purchased from VEC Technologies Inc. (Rensselaer, NY, USA). Briefly, cells were maintained in DMEM (supplemented with 10% FBS) and used between the 6th and 12th passage. Cells were grown to confluence in 6-well plates for western blot assays, real-time quantitative PCR (RT-qPCR) and liquid chromatography-tandem mass spectrometry (LC-MS/MS). Fatty acids and other reagents were dissolved in a medium supplemented with 10% FBS for treatments.

### 2.5. Fatty Acid Treatments

DHA or EPA was administered as a complex with fatty acid-free BSA, as previously described [19]. Briefly, DHA or EPA (0.3 mM each) was dissolved in ethanol (2.5 mL), gradually solubilized in an 8.4% BSA solution (14.3 mL) at 37 °C, and then in a serum-containing medium. 4-HHE was dissolved in dimethyl sulfoxide (DMSO) and then in a serum-containing medium.

### 2.6. Western Blot Analysis

Total protein samples from RAECs were prepared as previously descried [16], and were resolved by SDS-polyacrylamide gel electrophoresis (SDS-PAGE) before being transferred to polyvinylidene fluoride membranes. Membranes were incubated with antibodies against sEH, platelet endothelial cell adhesion molecule (PECAM-1), p38, phospho-p38, nuclear factor erythroid 2-related factor 2 (Nrf2), HO-1, and CYP2J2 or β-actin, and then incubated with HRP-conjugated secondary antibodies (followed by chemiluminescence reaction (PerkinElmer, Waltham, MA, USA)). The protein bands’ intensity was quantified using Scion image analysis software.

### 2.7. mRNA Extraction and Real-Time qPCR Analysis

Total RNA was extracted from the cells using the Total RNA Mini Kit (Bio-Rad, Hercules, CA, USA). Single-stranded cDNA was synthesized from 0.75 μg of total RNA using the Prime Script RT Reagent Kit (Takara Bio, Shiga, Japan), and endogenous genomic DNA was degraded by DNase I (Life Technologies). RT-qPCR experiments were carried out with the SYBR Green PCR master mix (Life Technologies) and the ABI 7500 Fast Real-Time PCR System (Applied Biosystems, Foster City, CA, USA). All quantitative data were normalized against the expression levels of 18S rRNA (18S). RT-qPCR conditions were 95 °C for 10 min, followed by 40 cycles of 95 °C for 15 s and 60 °C for 1 min. Primer sequences used were as follows: *epoxide hydrolase 2* (*EPHX2*) encoding sEH forward: CATTGTACTCCGTCCTGAAATGTCC, reverse: TGACCACAGTCTTCGATGTGTCC; 18S forward: TTCCGATAACGAACGAGACTCT, reverse: TGGCTGAACGCCACTTGTC.

### 2.8. Quantitative Analysis of 4-HHE in Biological Samples

4-HHE in RAECs was quantitatively analyzed using a modified LC-MS/MS procedure as described previously [20,21]. Briefly, a standard 4-HHE solution (Cayman Chemical Co., Ann Arbor, MI, USA) was used for the calibration curve. Solid phase extraction was done using a mixed-mode anion exchange solid-phase extraction (SPE) cartridge (Oasis MAX; Waters, Milford, MA, USA). An ACQUITY CSH C18 column (Waters) was used for separating 4-HHE. Electrospray ionization (ESI) was carried out with API4000 (operating in the positive ionization and selected reaction monitoring (SRM) mode). The SRM transitions for CHD-derivatized 4-HHE were m/z 284–216.

### 2.9. Quantitative Analysis of the Epoxyeicosatrienoic Acid/Dihydroxyeicosatrienoic Acid Ratio in RAECs

The EET/DHET ratio in RAECs was quantitatively analyzed using a modified LC-MS/MS procedure, as described previously [12]. Briefly, 400 μL of a chloroform/methanol (1:1, *v*/*v*) solution were added to RAECs in a 6-well plate. Isotonic sodium chloride solution (Otsuka Pharmaceutical Factory, Tokushima, Japan) was used as the surrogate matrix for the standard curve. To this surrogate matrix a standard solution of 11, 12-EET, 14, 15-EET, 11, 12-DHET and 14, and 15-DHET (Cayman Chemical Co.) diluted with methanol (0.05–100 ng/mL) was spiked. Calibration curve samples were extracted using the same procedure as cell lysate samples. For the extraction procedure, 2 mL of water was added to all samples for dilution. After vortexing, samples were added to SPE cartridges (Oasis HLB 3cc (60 mg) extraction cartridges, Waters). Ethyl acetate was used for elution, and this eluate was evaporated under a stream of nitrogen gas at 36 °C. After reconstitution with 30 μL of methanol, the aliquot was injected into an optimized LC-tandem MS system. LC was performed using an ACQUITY UPLC (Waters), and a Triple Quad 5500 tandem mass spectrometer (SCIEX, Foster City, CA, USA) was used as the detector. An analytical column (ACQUITY UPLC HSS T3, 1.8 μm, 2.1 mm × 150 mm; Waters) was used; the column temperature was maintained at 60 °C. Injected samples were eluted using water/acetonitrile/acetic acid (7:3:0.1, *v*/*v*/*v*) and acetonitrile/2-propanol (1:1, *v*/*v*), with a linear gradient at a total flow of 0.215 mL/min. For operation of Triple Quad 5500, electrostatic spray ionization in negative mode with selected reaction monitoring was selected. The selected reaction monitoring transition parameter database of Lipid Map was referred to for the selected precursor to product ion of m/z. Other parameters were adjusted to optimum values.

### 2.10. Statistical Analysis

The data were analyzed by SPSS 22.0 software (IBM SPSS, Armonk, NY, USA). Data are presented as the mean ± standard error of the mean (SEM). Statistical differences among more than three groups were analyzed by one-way analysis of variance (ANOVA), followed by Fisher’s protected least significant difference (PLSD) test. One-way ANOVA, followed by Tukey’s HSD post hoc analysis, was used to analyze four groups. A value of *p* < 0.05 was considered statistically significant.

## 3. Results

### 3.1. Fish Oil Treatment Improves Acetylcholine-Induced Vasodilation in Rat Aorta

**Figure 1:**
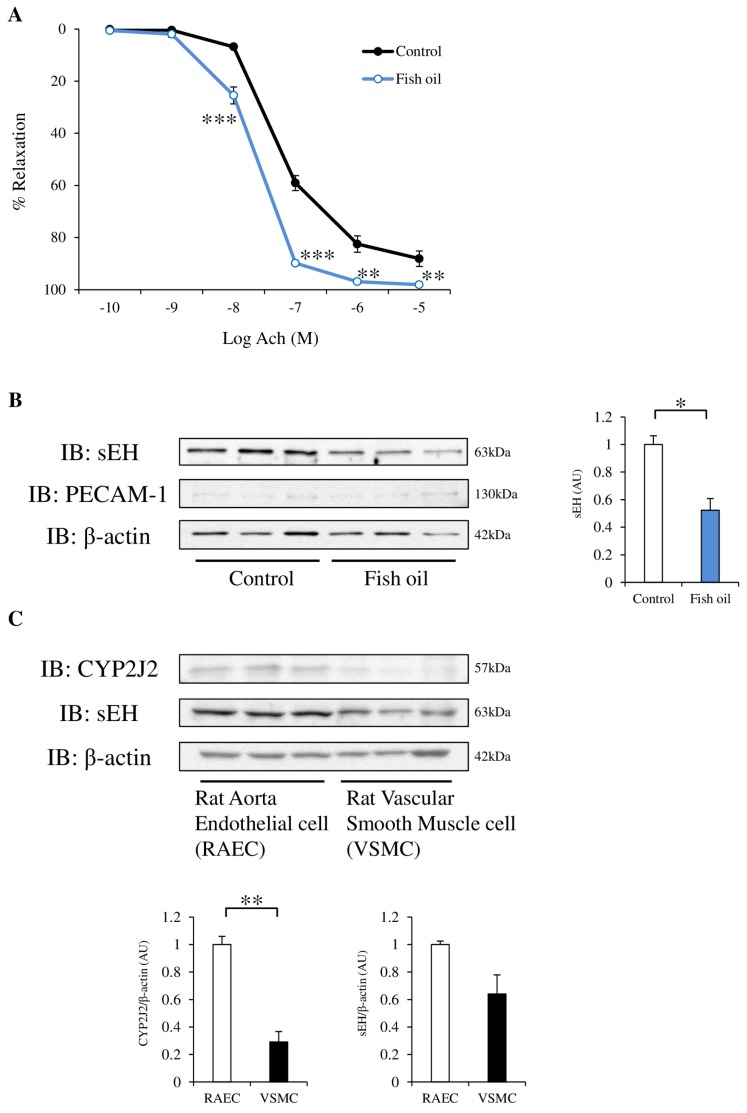
Effect of a fish oil diet on acetylcholine-dependent vasorelaxation in thoracic aortas. Protein expression of soluble epoxide hydrolase (sEH) in rat aortic strips, rat aorta endothelial cells (RAECs), and rat vascular smooth muscle cells. (**A**) Concentration-vasodilatory response curves induced by acetylcholine in aortic strips obtained from Sprague-Dawley (SD) rats, fed a control diet (black circles, black line) or fish oil diet (open circles, blue line) for 3 weeks. Results are expressed as the mean ± standard error of the mean (SEM) of six strips. (**B**) The protein expression of sEH, platelet endothelial cell adhesion molecule-1 (PECAM-1) (endothelial marker) and β-actin in aortic strips from control or fish oil diet-fed rats. *n* = 3. (**C**) The protein expression of cytochrome P450 2J2 (CYP2J2) and sEH in RAECs and rat vascular smooth muscle cells was examined by western blotting. *n* = 3. * *p* < 0.05, ** *p* < 0.01, *** *p* < 0.001, compared with control. IB: immunoblotting.

To confirm the effect of the fish oil diet on blood vessels, we tested the acetylcholine-induced vasodilation in rat aortic strips. Rats fed a fish oil diet for 3 weeks had significantly increased acetylcholine-induced vasodilation (Figure 1A). EET synthesis is induced by cytochrome P450 2J2 (CYP2J2). EETs are hydrolyzed to less active diols by the enzyme sEH. sEH protein expression in rat aortic strips was lower in the fish oil-fed group than in the control group (Figure 1B). The protein expression of PECAM-1, an endothelial marker, was weak both in the control and fish oil-fed rats. We investigated the protein expression level of CYP2J2 and sEH in RAECs and rat vascular smooth muscle cells, and found that the protein expression of both proteins was stronger in endothelial cells compared with vascular smooth muscle cells (Figure 1C).

### 3.2. DHA and EPA Downregulate sEH Protein Expression in RAECs

**Figure 2:**
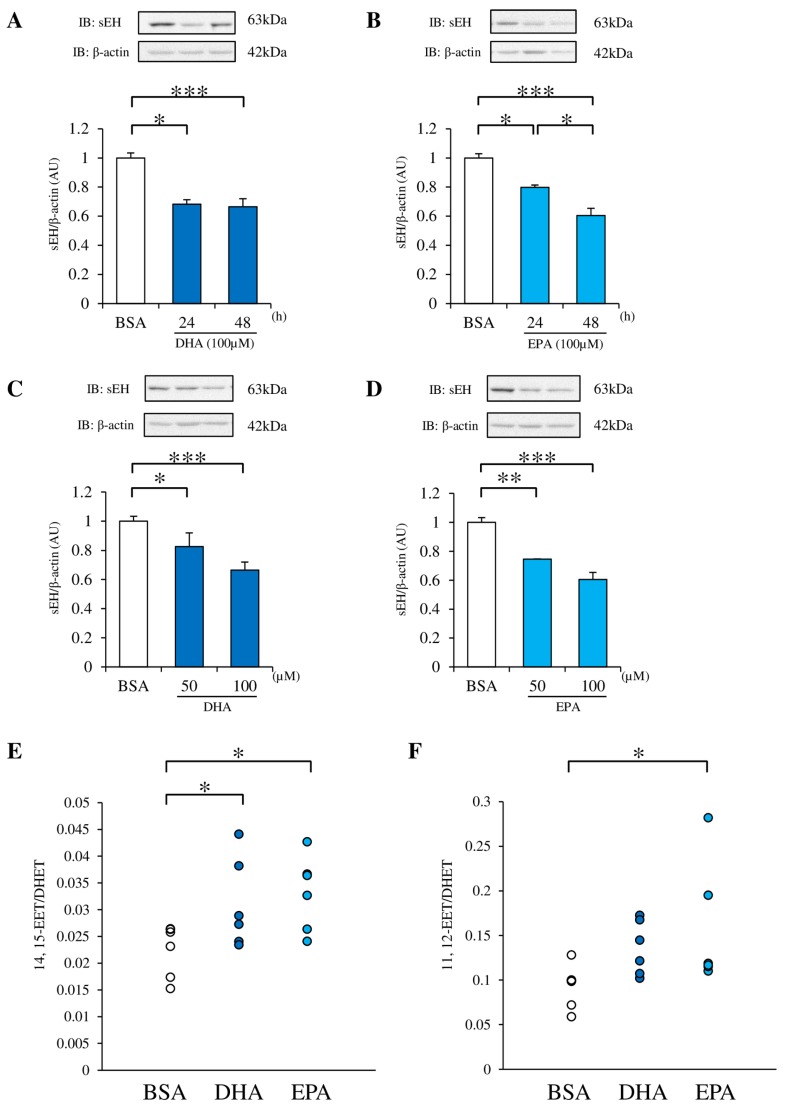
Effect of docosahexaenoic acid (DHA) or eicosapentaenoic acid (EPA) on sEH protein expression and epoxyeicosatrienoic acid/dihydroxyeicosatrienoic acid (EET/DHET) ratio. (**A**, **B**) The sEH protein expression in RAECs incubated with 100 μM DHA or EPA for 24 and 48 h was evaluated by western blotting and normalized against β-actin. (**C**, **D**) RAECs were incubated with 50 or 100 μM DHA or EPA for 48 h, and the sEH protein expression was examined. (E, F) RAECs were incubated with 100 µM DHA or EPA for 48 h, and the intracellular EET/DHET ratio was measured. 11, 12-EET, 14, 15-EET, 11, 12-DHET and 14, and 15-DHET were measured by liquid chromatography-tandem mass spectrometry (LC-MS/MS). Each value represents the mean ± SEM. (**A**) *n* = 25 (bovine serum albumin (BSA)), 3 (100 µM DHA, 24 h), 22 (100 µM DHA, 48 h); (**B**) *n* = 13 (BSA), 3 (100 µM EPA, 24 h), 10 (100 µM EPA, 48 h); (**C**) *n* = 32 (BSA), 10 (50 µM DHA, 48 h), 22 (100 µM DHA, 48 h); (**D**) *n* = 13 (BSA), 3 (50 µM EPA, 48 h), 10 (100 µM EPA, 48 h); (E, F) *n* = 6. * *p* < 0.05, ** *p* < 0.01, *** *p* < 0.001, compared with control. BSA: Fatty acid-free bovine serum albumin.

To explore the mechanism of the improvement in endothelial function by fish oil treatment, we used cultured endothelial cells. EPA or DHA treatment for 24 and 48 h reduced sEH protein expression in RAECs, compared with BSA treatment (Figure 2A,B). Furthermore, DHA and EPA reduced sEH protein expression in a dose-dependent manner (Figure 2C,D). To test the significance of the DHA or EPA-induced sEH suppression, the EET/DHET ratio was directly measured in RAECs by LC-MS/MS. A significant increase was observed in the EET/DHET ratios after treatment with DHA or EPA (Figure 2E,F).

### 3.3. 4-HHE Downregulates sEH Protein Expression

**Figure 3:**
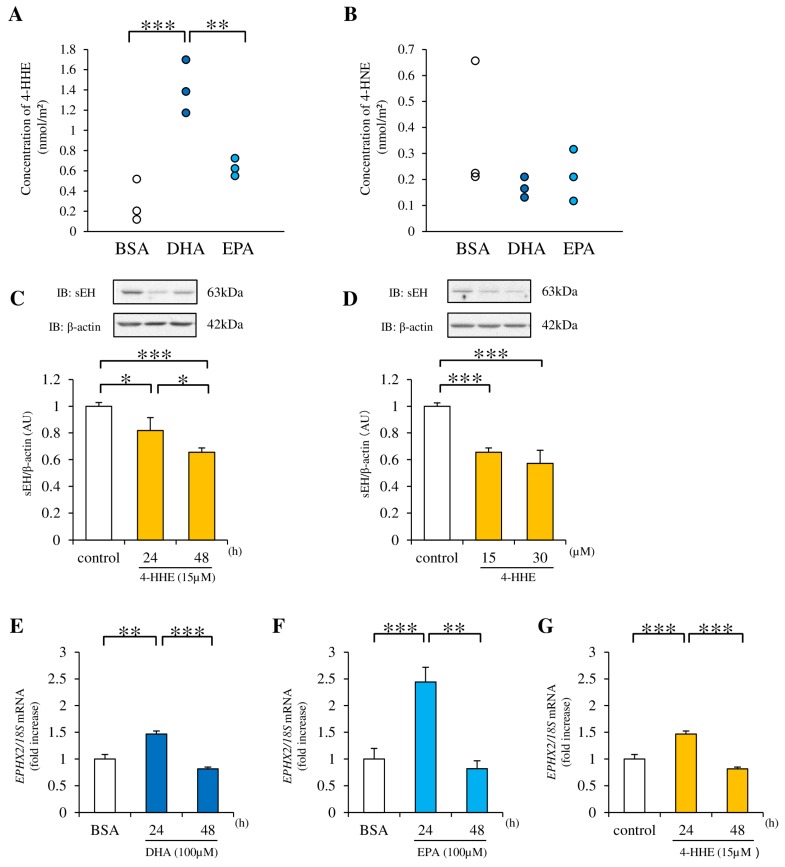
Effect of DHA and EPA on intracellular 4-hydroxy hexenal (4-HHE) levels. Effect of 4-HHE on sEH expression. (**A**, **B**) The concentration of intracellular 4-HHE in RAECs treated with 100 µM DHA or EPA was measured by LC-MS/MS analysis. (**C**, **D**) RAECs were incubated with 15 or 30 µM 4-HHE for 24 and 48 h, and the protein expression of sEH and β-actin was examined by western blotting. (E–G) RAECs were incubated with 100 µM DHA or EPA, or 15 µM 4-HHE for 24 and 48 h. The relative mRNA level of *epoxide hydrolase 2 (EPHX2)* was quantified using real-time quantitative PCR (RT-qPCR). Each value represents the mean ± SEM. (**A**, **B**) *n* = 3; (**C**) *n* = 19 (control), 3 (15 µM 4-HHE, 24 h), 16 (15 µM 4-HHE, 48 h); (**D**) *n* = 25 (control), 16 (15 µM 4-HHE, 48 h), 9 (30 µM 4-HHE, 48 h); (E–G) *n* = 6 (BSA), *n* = 3 (100 µM DHA or EPA, 15 µM 4-HHE, 24 or 48 h). * *p* < 0.05, ** *p* < 0.01, *** *p* < 0.001, compared with control.

The concentration of free 4-HHE (a lipid peroxide) in RAECs was measured by LC-MS/MS after a 7 h incubation with *n*-3 PUFAs. Compared with the control (BSA), a greater increase in the 4-HHE concentration was observed after treatment with DHA than with EPA (Figure 3A). In contrast, the concentration of 4-hydroxynonenal (4-HNE), another lipid peroxide derived from *n*-6 PUFAs, tended to be lower than the control after treatment with DHA or EPA (Figure 3B). To test the role of 4-HHE in the regulation of EET metabolism, 4-HHE was directly added to the medium. This treatment suppressed sEH protein expression in RAECs (Figure 3C,D). In contrast, the *EPHX2* mRNA (encoding sEH) expression was increased at 24 h by DHA, EPA, or 4-HHE, and returned to baseline at 48 h (suggesting posttranslational modification in this process (Figure 3E–G)).

### 3.4. NAC Pretreatment Inhibits the Decrease in sEH Protein Induced by DHA, EPA or 4-HHE

**Figure 4:**
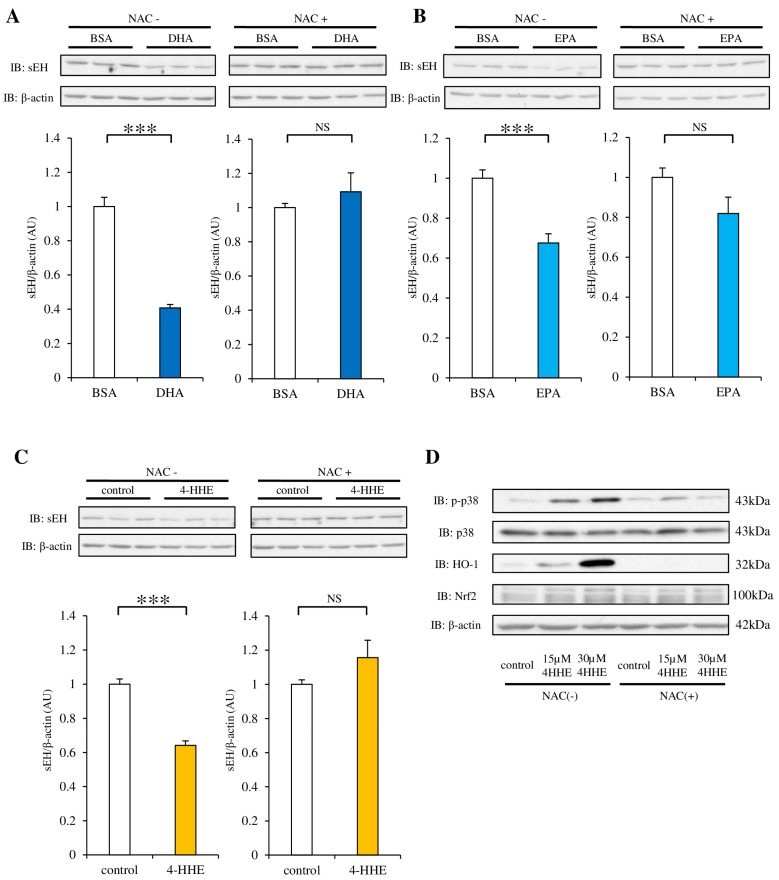
Effect of antioxidants on DHA, EPA, or 4-HHE-suppression of sEH protein expression. (**A**–**C**) RAECs were pretreated with N-acetyl-l-cysteine (NAC; 10 mM) for 1 h, and then with DHA (100 µM), EPA (100 µM), or 4-HHE (15 µM) for 48 h. The protein expression of sEH and β-actin was evaluated by western blotting. Each value represents the mean ± SEM. *n* = 7. (**D**) The protein expression of p38 kinase (p38), phospho-p38 (p-p38), heme oxygenase 1 (HO-1), nuclear factor erythroid 2-related factor 2 (Nrf2), and β-actin (in cells treated with 15 or 30 µM 4-HHE for 48 h) was determined by western blotting. * *p* < 0.05, ** *p* < 0.01, *** *p* < 0.001, compared with control. NS, no significant difference.

We have previously reported the protective effect of 4-HHE (derived from *n*-3 PUFA) on the endothelium via Nrf2, a key transcription factor for antioxidants and oxidative stress [16]. To evaluate the role of oxidative stress induced by DHA, EPA, or 4-HHE, we used NAC (an antioxidant that mimics glutathione), which can bind to lipid peroxide. The decrease in sEH protein expression after DHA, EPA, or 4-HHE treatment was abolished by pretreatment with 10 mM NAC (Figure 4A–C). NAC treatment also abolished the 4-HHE-induced HO-1 protein expression and p38 phosphorylation (Figure 4D), suggesting that oxidative stress mediated by *n*-3 PUFAs is upstream of Nrf2 and p38 kinase.

### 3.5. siRNA Against Nrf2 Inhibits the Decrease in sEH Protein Expression Induced by DHA, EPA, or 4-HHE

**Figure 5:**
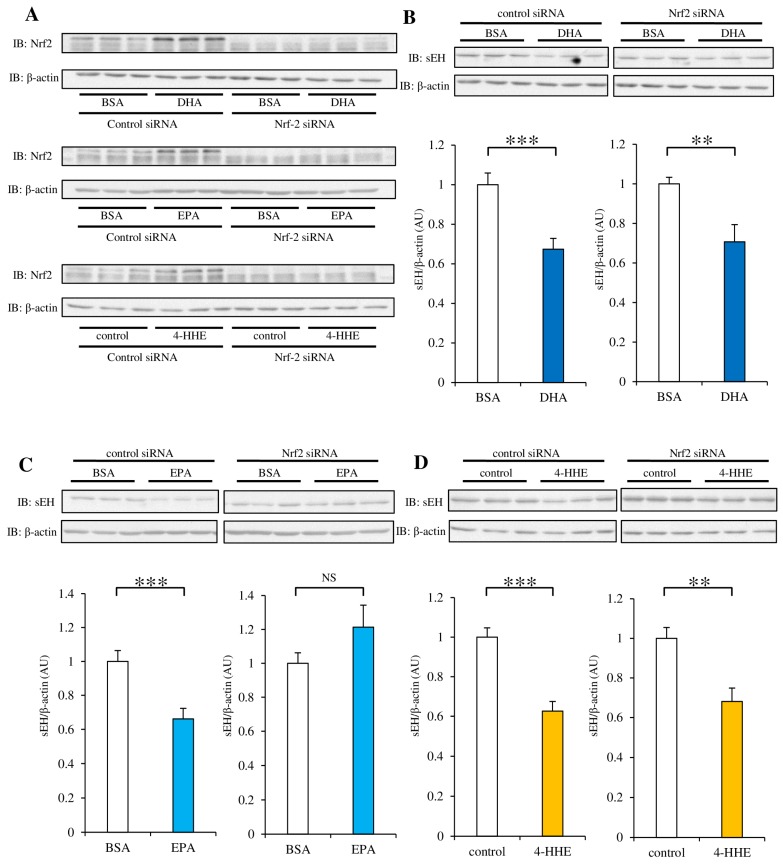
Effect of Nrf2-knockdown on DHA, EPA, or 4-HHE-suppression of sEH protein expression. RAECs were transfected with *Nrf2* Small interfering RNA (siRNA) or control siRNA. After 24 h, the cells were incubated with DHA (100 µM), EPA (100 µM), or 4-HHE (15 µM) for another 48 h. Whole cell lysates were subjected to western blot analysis. (**A**) Nrf2 and β-actin expression was determined by western blotting. (**B**–**D**) sEH and β-actin expression levels were determined by western blotting. Each value represents the mean ± SEM. *n* = 6-10. * *p* < 0.05, ** *p* < 0.01, *** *p* < 0.001, compared with control. NS, no significant difference.

Next, we investigated the role of Nrf2 in sEH protein expression suppressed by *n*-3 PUFAs. To evaluate the efficiency of siRNA against Nrf2, we tested the protein expression of Nrf2 in RAECs. We found that incubation with DHA, EPA, or 4-HHE for 48 h stimulated the expression of the Nrf2 protein, but siRNA treatment against Nrf2 efficiently suppressed its expression in RAECs (Figure 5A). The effect of Nrf2-knockdown on the protein expression of sEH varied among DHA, EPA, and 4-HHE (Figure 5B–D). These results suggest that Nrf2 activation by DHA or 4-HHE may not have a significant role in the regulation of sEH protein expression. However, Nrf2 activation by EPA may contribute to the regulation of sEH expression.

### 3.6. Pretreatment with the p38 Kinase Inhibitor SB203580 Rescues the Decrease in sEH Protein Expression Induced by DHA, EPA or 4-HHE

**Figure 6:**
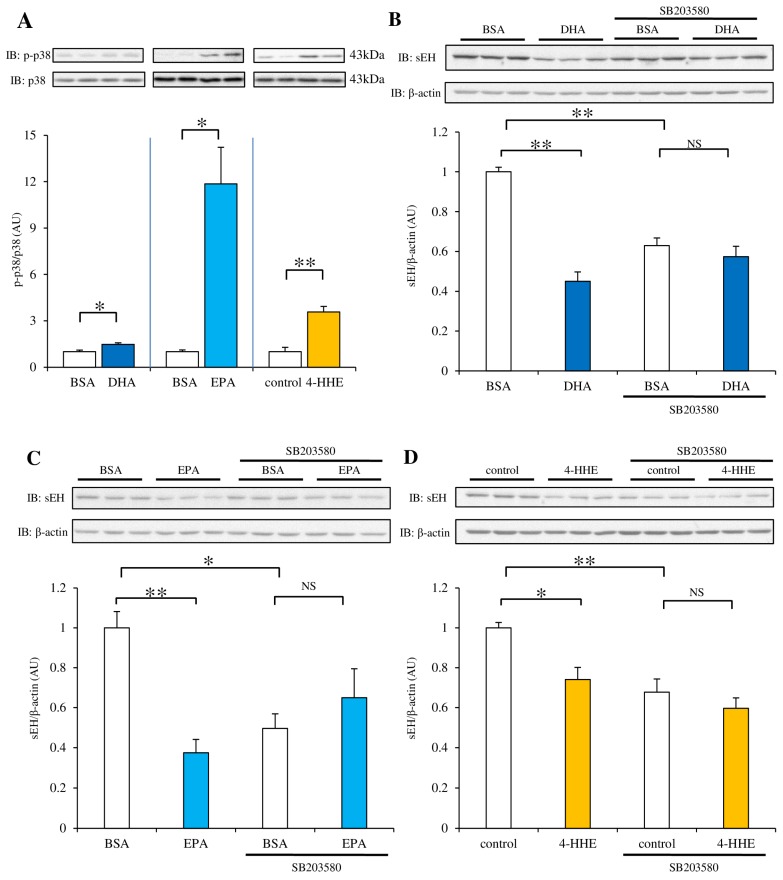
Effect of the p38 kinase inhibitor (SB203580) on DHA, EPA, or 4-HHE-suppression of sEH protein expression. (A) RAECs were incubated with DHA (100 µM), EPA (100 µM), or 4-HHE (15 µM) for 48 h. p-p38 and p38 levels were determined by western blotting. *n* = 3. (B–D) RAECs were treated with the p38 kinase inhibitor SB203580 (10 µM) for 30 min before control, DHA (100 µM), EPA (100 µM), or 4-HHE (15 µM) incubation. sEH and β-actin protein levels were determined by western blotting. Each value represents the mean ± SEM. *n* = 6–7. (B–D) One-way ANOVA followed by Tukey’s HSD post hoc analysis. * *p* < 0.05, ** *p* < 0.01. NS, no significant difference.

We examined the phosphorylation of p38 kinase after treatment with DHA, EPA, or 4-HHE. Incubation with DHA for 48 h resulted in a 1.5 fold, slight (but significant) increase in p38 kinase phosphorylation. Incubation with EPA and 4-HHE showed a much stronger effect (at least at 48 h) compared with DHA (Figure 6A). To test the role of p38 activation in sEH protein levels, we pretreated the cells with SB203580 (a p38 inhibitor), followed by DHA, EPA, or 4-HHE treatment. As expected, the sEH protein levels reduced after DHA treatment. In addition, the sEH protein levels also decreased after SB203580 treatment alone, suggesting basal p38 kinase activity may increase sEH protein levels (Figure 6B). There was no significant difference between treatment with DHA + SB203580 and SB203580 alone, suggesting SB203580 pretreatment inhibited the DHA-induced decrease in sEH protein expression (although SB203580 treatment itself decreased basal sEH levels (Figure 6B)). Both EPA and 4-HHE showed a similar result to DHA (Figure 6C,D).

## 4. Discussion

The three important findings of this study are: (1) *n*-3 PUFAs decreased sEH protein expression in rat aortic strips and endothelial cells; (2) 4-HHE (derived from *n*-3 PUFA) also decreased sEH protein expression, which was inhibited by the antioxidant NAC; and (3) p38 kinase activation by oxidative stress may be downstream of the 4-HHE-induced decrease in sEH protein expression.

The *n*-3 PUFA-induced decrease in sEH protein expression in rat aortic strips and rat endothelial cells is consistent with observations in the liver of *n*-3 PUFA-treated rats [22]. Moreover, intervention with both alpha linoleic acid and a fish-based diet in patients with hypertension [23] or type 2 diabetes [12] resulted in an increased EET/DHET ratio in the plasma, with improved endothelial function. Considering the increased sEH expression in the mesenteric artery of obese Zucker rats [24], fish oil treatment may improve endothelial function by decreasing sEH expression.

Cardiovascular risk factors are associated with increased levels of reactive oxygen species, and cause endothelial dysfunction mainly through eNOS uncoupling [25]. We have reported that 4-HHE, a lipid metabolite of *n*-3 PUFA, is a potential activator of the Nrf2 pathway, which has a significant role in antioxidative stress and endothelial function [16]. We have been particularly interested in 4-HHE because we found that plasma 4-HHE levels were increased upon fish-based diet intervention in patients with type 2 diabetes [12]; 4-HHE levels in rat aortic strips (incubated for 6 h with DHA or EPA) were increased [19]. In this study, the antioxidant NAC significantly suppressed the *n*-3 PUFA-induced decrease in sEH expression, suggesting that *n*-3 PUFAs may increase the EET concentration (through inhibition of sEH expression) by inducing oxidative stress. siRNA against Nrf2 abolished the decrease in sEH protein expression induced by EPA, but not by DHA or 4-HHE. This is partially consistent with our previous observation in Nrf2-knockout mice, in which 3 weeks of a fish oil diet failed to improve the acetylcholine-induced endothelial dilation [16]. In the current study, EPA activated p38 kinase stronger than DHA (Figure 6A). The difference between DHA and EPA may be explained by an Nrf2-dependent antioxidative pathway. In addition to the Nrf2-dependent antioxidative pathway, 4-HHE derived from DHA and EPA may have a direct effect on sEH stability. It has been reported that protein degradation was increased by 4-HNE (derived from *n*-6 PUFA) via a proteasomal pathway [26]. To test this possibility, we examined the effect of MG132, a proteasomal inhibitor, and found that it failed to protect sEH from protein degradation.

Inhibition of p38 kinase diminished the effect of *n*-3 PUFAs and 4-HHE on sEH protein expression. As previously demonstrated, DHA, EPA, and 4-HHE stimulated p38 kinase phosphorylation (although it was low in DHA-treated cells at 48 h (Figure 6A)); this is in agreement with a previous study, showing that oxidative stress induced p38 kinase activation in vascular endothelial cells [27]. A recent study has shown that an increase in specificity protein 1 (Sp1) expression was suppressed by SB203580, which also inhibited the decrease in sEH expression under high glucose (implying the involvement of the p38 kinase pathway in the increase in nuclear Sp1 expression) [28]. It has been shown that oxidative stress stimulated the binding of Sp1 to GC-rich regions of the *caveolin-1* promoter, through p38 kinase signaling [29]. However, we found increased *EPHX2* mRNA levels in DHA or EPA-treated cells, suggesting that p38-mediated activation of Sp1 is not the mechanism responsible for the decreased expression of sEH. Instead, we think that moderate oxidative stress induced by *n*-3 PUFAs may decrease sEH protein levels via p38 kinase activation.

Dyslipidemia, hypertension, hyperglycemia, smoking, and hypomagnesemia are all potential causes of oxidative stress and endothelial dysfunction [2,30]. Vitamin E and other anti-oxidative medications have been shown to improve endothelial function [31]. However, exercise causes oxidative stress but improves endothelial function [32]. The strength of our study is that we found that *n*-3 PUFAs work like exercise on blood vessels. Further experiments are necessary to elucidate the interactions among oxidative stress, inflammation, and endothelial function in the progression of atherosclerosis.

This study has some limitations. First, we did not fully identify the mechanism; i.e., we did not reveal the direct target of p38 kinase or 4-HHE in the regulation of sEH protein expression. Second, the rat aorta results may not explain the phenotype in human peripheral arteries, as previous studies have reported that the effects of eNOS and EDHF on endothelial function vary among different organs and species [2]. Because a previous report has shown increased eNOS expression in the aortas of *n*-3 PUFA-fed rats [17], our findings in this study on sEH regulation may partially explain the fish oil-induced improvement in endothelial function. However, any generalizations must be made with caution. Third, we did not analyze the role of vasodilation (induced by *n*-3 metabolites) derived from the sEH pathway. Not only arachidonic metabolites, but also *n*-3 PUFA, mediated epoxide (which is regulated by sEH); this may play a significant role in vasodilation [33].

## 5. Conclusions

DHA and EPA inhibited sEH protein expression, probably through a 4-HHE-p38 kinase pathway. These findings may explain the protective effect of *n*-3 PUFAs on blood vessels.

## Abbreviations

sEH	Soluble epoxide hydrolase
EET	epoxyeicosatrienoic acid
PECAM-1	platelet endothelial cell adhesion molecule-1
Nrf2	nuclear factor erythroid 2-related factor 2
4-HHE	4-hydroxy hexenal
PUFAs	polyunsaturated fatty acids
DHA	docosahexaenoic acid
EPA	eicosapentaenoic acid
HO-1	heme oxygenase-1
p38	p38 kinase
